# Muscle Ultrasound Shear Wave Elastography as a Non-Invasive Biomarker in Myotonia

**DOI:** 10.3390/diagnostics11020163

**Published:** 2021-01-23

**Authors:** Cornelius Kronlage, Alexander Grimm, Alyssa Romano, Jan-Hendrik Stahl, Pascal Martin, Natalie Winter, Justus Marquetand

**Affiliations:** Department of Neurology and Epileptology, University Hospital Tübingen and Hertie Institute for Clinical Brain Research, University of Tuebingen, 72076 Tuebingen, Germany; alexander.grimm@med.uni-tuebingen.de (A.G.); alyssa.romano@student.uni-tuebingen.de (A.R.); jan-hendrik.stahl@med.uni-tuebingen.de (J.-H.S.); pascal.martin@med.uni-tuebingen.de (P.M.); natalie.winter@med.uni-tuebingen.de (N.W.); justus.marquetand@med.uni-tuebingen.de (J.M.)

**Keywords:** ultrasound elastography, shear-wave elastography, muscle, myotonia

## Abstract

Myotonia, i.e., delayed muscle relaxation in certain hereditary muscle disorders, can be assessed quantitatively using different techniques ranging from force measurements to electrodiagnostics. Ultrasound shear wave elastography (SWE) has been proposed as a novel tool in biomechanics and neuromuscular medicine for the non-invasive estimation of muscle elasticity and, indirectly, muscle force. The aim of this study is to provide ‘proof-of-principle’ that SWE allows a quantitative measurement of the duration of delayed muscle relaxation in myotonia in a simple clinical setting. In six myotonic muscle disorder patients and six healthy volunteers, shear wave velocities (SWV) parallel to the fiber orientation in the flexor digitorum superficialis muscle in the forearm were recorded with a temporal resolution of one per second during fist-clenching and subsequent relaxation; the relaxation time to 10% of normalized shear wave velocity (RT_0.1_) was calculated. Forty-six SWE imaging sequences were acquired, yielding a mean RT_0.1_ of 7.38 s in myotonic muscle disorder patients, significantly higher than in healthy volunteers (1.36 s), which is comparable to data obtained by mechanical dynamometry. SWV measurements during the baseline relaxation and voluntary contraction phases did not differ significantly between groups. We conclude that SWE is a promising, non-invasive, widely available tool for the quantitative assessment of myotonia to aid in diagnosis and therapeutic monitoring.

## 1. Introduction

Myotonia describes delayed muscle relaxation after activation. It is the eponymous symptom and clinical sign of myotonic muscle disorders, causing disability and reduction in quality of life [1,2]. Hand-grip is a typical trigger of a myotonic reaction, i.e., the—functionally highly relevant—finger flexor muscles are often affected in myotonic muscle disorders. Pathophysiologically, there is hyperexcitability of myocytes due to genetic abnormalities. Various medications, many acting as sodium channel blockers, have been investigated for symptomatic therapy and found to alleviate myotonia [3,4,5,6].

However, there is no gold-standard outcome measure for the assessment of myotonia or the effect of medication. Previous clinical studies used the following options: (1) patient-reported outcomes, such as the Myotonia Behaviour Scale [7]; (2) clinical observation and timing of myotonia after closing eyes or clenching a fist, as well as the presence of percussion myotonia [3,5]; (3) quantitative measurement of the increased relaxation time of finger flexors with a dynamometer [8,9,10,11]; (4) functional tests of the lower extremities such as the Timed Up And Go or 14 Step Stair Test [6]; and (5) electrophysiological tests such as electromyographical relaxation time [3] or the maximal post-exercise decrement in compound muscle action potential after short and long exercise [5].

These approaches each have different advantages in terms of availability, reliability, and validity, but also different drawbacks, e.g., functional tests may be confounded by other disabilities, dynamometry requires specialised equipment, and motor nerve stimulation in electrophysiological testing causes discomfort.

Ultrasound has found wide application in the diagnosis of neuromuscular diseases. It is nowadays a well-established technique supplementing electrodiagnostic tests for neurologists and electrophysiologists in routine clinical settings such as the diagnostic evaluation of myopathies [2,12]. Therefore, ultrasound may serve as a simple, pragmatic, and easily available tool for the assessment of myotonia. The duration of percussion myotonia in the thenar eminence has been successfully quantified using B-mode ultrasound [13]. Whereas B-mode ultrasound reveals structure and motion, ultrasound elastography enables the non-invasive estimation of mechanical properties of tissue in vivo [14]. Various techniques exist; some, e.g., so-called strain elastography, are based on the measurement of tissue displacement in response to an applied force. More indirectly, the velocity of shear waves can be measured and processed to infer mechanical elasticity; in research systems, shear wave attenuation and frequency dispersion are also used to calculate tissue viscosity [15]. Two-dimensional (2D) acoustic radiation force-based shear wave elastography (SWE), as used in this work and widely available on clinical ultrasound systems, relies on real-time tracking of acoustically induced shear waves that propagate perpendicularly to the main transducer transmission direction. SWE generates quantitative maps of shear wave velocity or shear modulus (which is quadratically related to the former). Various clinical applications for SWE have been established, e.g., non-invasive estimation of liver fibrosis [16] or differentiation of breast lesions [17,18].

Muscle elasticity as measured by SWE is affected, on the one hand, by structural changes: for instance, elasticity appears to be decreased in myositis [19,20] and increased in Duchenne muscle dystrophy [21,22]. On the other hand, shear modulus in the longitudinal direction—parallel to muscle fibres—has been shown ex vivo and in vivo to be linearly related to passive and active muscle forces [23,24,25], such as induced by different joint angles or muscle activation. Therefore, SWE represents a tool for the non-invasive estimation of individual muscle force.

In this work, we aim to show that SWE in a clinical ultrasound system with a temporal resolution of one second allows the observation of myotonia. Myotonia typically occurs in the finger flexor muscles in the forearm and, consequently, during hand-grip movements. Hence, we devised a protocol to measure the longitudinal shear wave velocity (SWV) as a proxy for muscle force in the flexor digitorum superficialis during a standardised fist-clenching manoeuvre and subsequently calculated the muscle relaxation time. This highlights the potential of ultrasound SWE as a simple, pragmatic, and easily available alternative for the assessment of myotonia and as an extension of clinical neurophysiology and neuromuscular ultrasound for functional tests.

## 2. Materials and Methods

### 2.1. Participants

Six patients with a known myotonic reaction (i.e., delayed muscle relaxation) were recruited from our outpatient clinic for muscle disorders (age 22–53 years, mean 35.2 years, 3 female). The underlying myotonic disease was genetically confirmed in five out of six patients (see Table 1, patient characteristics). In the remaining patient, the result of genetic testing was not yet available at the time the study was conducted. Nevertheless, there was no clinical or electrophysiological doubt about the presence of a myotonic disease, since typical myotonic discharges were found on needle electromyography (EMG) and delayed muscle relaxation was observed. The supplemental video depicts hand-grip myotonia being elicited in this patient (no. 6).

For comparison of the delayed muscle relaxation, six healthy volunteers (age 25–66 years, mean 35.7 years, 3 female) were recruited. They had no history or clinical signs of neuromuscular disease.

All participants were >18 years of age and all provided informed consent. The study was approved by the local ethics review committee (University of Tuebingen) and conducted according to the declaration of Helsinki.

### 2.2. Ultrasound Imaging Protocol

Ultrasound imaging was performed with a Canon Aplio i800 system (Canon Medical Systems, Neuss, Germany) using a 4–18 MHz linear transducer (i18LX5/PLI-1205BX, Canon Medical Systems, Neuss, Germany). This system implements a comb-push shear wave elastography (SWE) technique [26]. Alfuraih and coworkers compared a comb-push SWE system with a more widely used system (Supersonic Imagine Aixplorer), finding comparable results in muscle [27]. For B-mode and SWE imaging, the machine’s presets for musculoskeletal imaging were used, with the following adjustments in SWE mode: minimum possible size of the region of interest (ROI), frame rate: 2 (maximum setting, resulting in a frame rate of 1/s), time smoothing: 0 (no time averaging), map type: speed (display of the shear wave velocity in meters per second).

**Figure 1:**
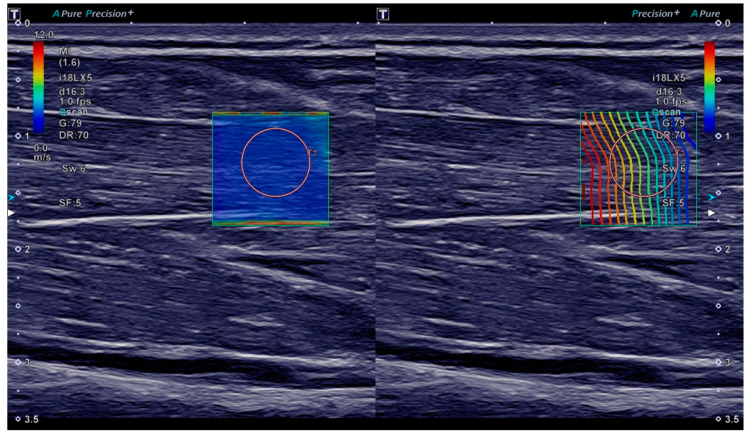
Ultrasound B-mode and shear wave elastography (SWE) imaging of the flexor digitorum superficialis muscle. On both sides of the screen, the same B-mode image of the muscle in the longitudinal plane is displayed. On the left, a shear wave velocity map is overlaid with the colour scale ranging from 0.0 m/s (blue) to 12.0 m/s (red). On the right, a proprietary shear wave propagation display is shown, meant to depict the shear wave wavefront. The orange circles are the shear wave velocity measurement ROI placed after acquisition.

For ultrasound measurements, the participants were sitting, with their elbow flexed approximately 90 degrees and their forearm supinated and placed on the thigh. We always examined the right arm. The ultrasound transducer was held by hand and positioned so as to obtain a longitudinal B-mode view of the flexor digitorum superficialis muscle in the middle third of the forearm, parallel to the muscle fibres. We targeted the flexor digitorum superficialis muscle because hand-grip is classically affected in myotonia and because of the methodical resemblance to the use of hand-grip dynamometers in myotonia. As SWE measurements are known to become more noisy with increasing depth [28,29], we measured the superficial instead of the deep finger flexor muscle. A square ROI in the flexor digitorum superficialis muscle was selected in the B-mode image for continuous shear wave elastography (SWE) with a frame rate of 1/s (Figure 1). Only minimal, constant contact force was applied with the transducer as is generally recommended [14,29].

**Figure 2:**
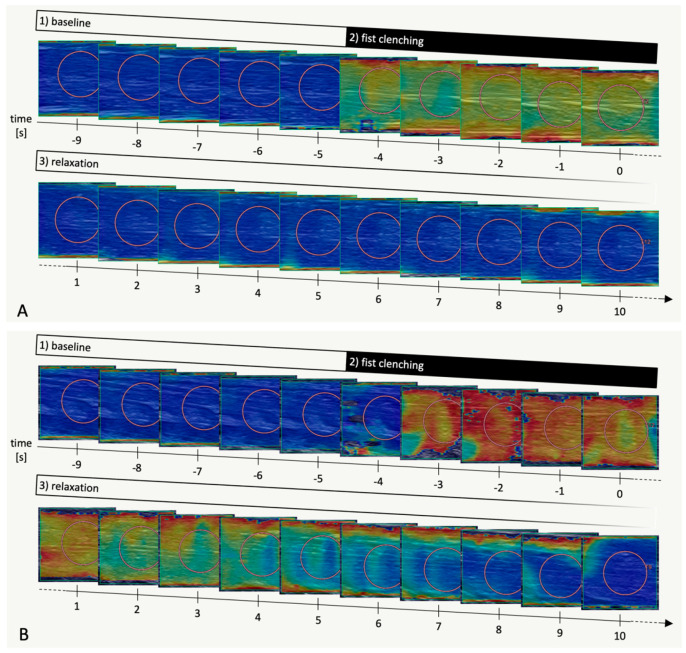
Representative SWE imaging sequences (flexor digitorum superficialis muscle) in a healthy volunteer (**A**) and in a myotonic muscle disorder patient (**B**), respectively. B-mode images in grey scale overlaid with shear wave velocity data in a colour scale ranging from 0.0 m/s (blue) to 12.0 m/s (red). Each image corresponds to one second. Image acquisition was continued beyond time = 10 s but the images are not shown here for the sake of clarity. Orange circles are shear wave velocity measurement ROIs placed after acquisition.

Participants were instructed to perform multiple fist-clenching manoeuvres one after another. There was no defined resting period or ‘warm-up’ procedure. Each manoeuvre was recorded in what is hence termed an SWE ‘imaging sequence’, with each sequence consisting of three phases (as illustrated in Figure 2):

(1) With participants having been instructed to relax their forearm and hand, SWE imaging was performed for 5 s (time −9 s to −5 s) for baseline measurements. At this stage, SWE quality was assessed visually in the shear wave velocity and propagation maps—if insufficient, the transducer was repositioned and imaging was restarted. Only if a homogeneously low (<2.5 m/s) shear wave velocity was observed in multiple successive images without artifacts (as in Figure 1), the imaging sequence was continued, keeping the transducer position in place.

(2) Participants were then instructed to clench their fist with maximum force for 5 s (time −4 s to 0 s) and

(3) to immediately relax their forearm and hand again without actively extending their fingers. SWE imaging continued until the shear wave velocity map on visual assessment had approximately returned to baseline for at least 3 successive images or—for controls—for at least 15 s.

In some cases, the fist-clenching manoeuvre caused inadvertent movement of the transducer, leading to low SWE quality in the relaxation phase with obvious artefacts or shear wave velocities not returning to baseline even after longer observation periods. If obvious, these imaging sequences were discarded at the time of acquisition. A second check was performed in data processing (as described below).

Overall, the ultrasound examination session lasted 30–45 min for each participant. The maximum possible number of imaging sequences was acquired in this time, which depended on the time necessary for transducer placement and repeated or excluded measurements due to insufficient SWE quality.

### 2.3. Data Processing

**Figure 3:**
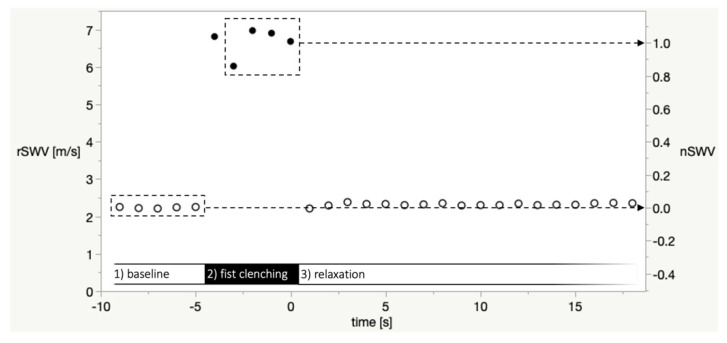
Plot of raw shear wave velocity values (rSWV, in m/s, left *y*-axis) of a representative SWE imaging sequence in a healthy volunteer. Open circles: Baseline and relaxation phase, closed circles: fist clenching phase. The dashed boxes depict the baseline phase measurements and the fist clenching phase images (time −3 to 0). The mean of these rSWV was defined as nSWV = 0 or nSWV = 1, respectively, in the per-sequence normalization of values as shown in the right *y*-axis. The value at time = −4 was excluded because in some imaging sequences, the participants’ reaction to the instruction to clench their fist was delayed.

On the ultrasound device, a circular measurement ROI (size setting: 6) was manually placed in each SWE map. When there was a heterogeneous distribution of shear wave values, the measurement ROI was placed so as to obtain low values with low variability (as determined by the standard deviation of values in the measurement ROI displayed on-screen). For each image—corresponding to one second in time—the mean shear wave velocity value in the measurement ROI in meters per second (termed raw shear wave velocity, rSWV) was used for further processing and analysis. Figure 3 is a plot of rSWV values of a representative SWE imaging sequence in a healthy volunteer.

As mentioned above, in some imaging sequences (both healthy volunteers and patients), unavoidable displacements of the hand-held ultrasound transducer relative to the forearm during the fist-clenching manoeuvre caused imaging artifacts. In these cases, we observed that shear wave velocities did not return to baseline even after extended observation periods. Thus, we only included imaging sequences in the final analysis if the last five images were comparable to the baseline (criteria: difference mean rSWV of the last five images to mean baseline rSWV <0.5 m/s and standard deviation of the nSWV of the last five images <0.2 m/s). Examples of imaging sequences with insufficient quality excluded at this stage are shown in Supplementary Materials Figure S1. Overall, 10 out of 56 imaging sequences (8 in myotonic muscle disorder patients, 2 in healthy volunteers) were excluded during data processing according to criteria described above (examples are shown in Supplemental Figure S1).

The following mean values were calculated for each imaging sequence: rSWV_mean baseline phase_ as the mean rSWV of all baseline phase images (time −9 s to −5 s). rSWV_mean fist clenching_ as the mean rSWV of fist clenching phase images (time −3 s to 0 s); time −4 was not included because some participants’ reaction to the verbal instruction to clench their fist was delayed (see discussion section).

Subsequently, for better comparability of different imaging sequences and calculation of relaxation times (RT), normalized shear wave velocities (nSWV) were calculated:

$$\mathrm{nSWV}=\frac{\left(\mathrm{rSWV}-{\mathrm{rSWV}}_{\mathrm{mean}\;\mathrm{baseline}\;\mathrm{phase}}\right)}{({\mathrm{rSWV}}_{\mathrm{mean}\;\mathrm{fist}\;\mathrm{clenching}\;\mathrm{phase}\;}-\;{\mathrm{rSWV}}_{\mathrm{mean}\;\mathrm{baseline}\;\mathrm{phase}}).\;}$$

This results in nSWV_mean baseline phase_ = 0 and nSWV_mean fist clenching phase_ = 1, as illustrated in Figure 3.

The relaxation time 0.1 (RT_0.1_) was defined as the time of the first measurement in the relaxation phase where nSWV < 0.1 (examples in Supplemental Figure S2).

### 2.4. Statistical Analysis

For statistical analysis, the software JMP 15.1 (SAS, Cary, NC, USA) was used. For descriptive statistics, the median, mean, and standard deviation are stated.

RT_0.1_, rSWV_mean baseline phase_ and rSWV_mean fist clenching phase_ were compared between groups (healthy volunteers vs. myotonic muscle disorder patients). As sample sizes were small and normality could not be assumed, we calculated a Wilcoxon rank sum test. The significance level was set at *p* < 0.05. For the differentiation between groups according to RT_0.1_, a receiver operating characteristic (ROC) curve analysis was performed. Sensitivity and specificity are stated for the threshold with maximal sensitivity-(1-specificity).

## 3. Results

In total, 46 SWE imaging sequences of sufficient quality were acquired, 21 in myotonic muscle disorder patients and 25 in healthy volunteers serving as controls; four sequences median per participant, range one to seven in patients, range two to six in healthy volunteers.

**Figure 4:**
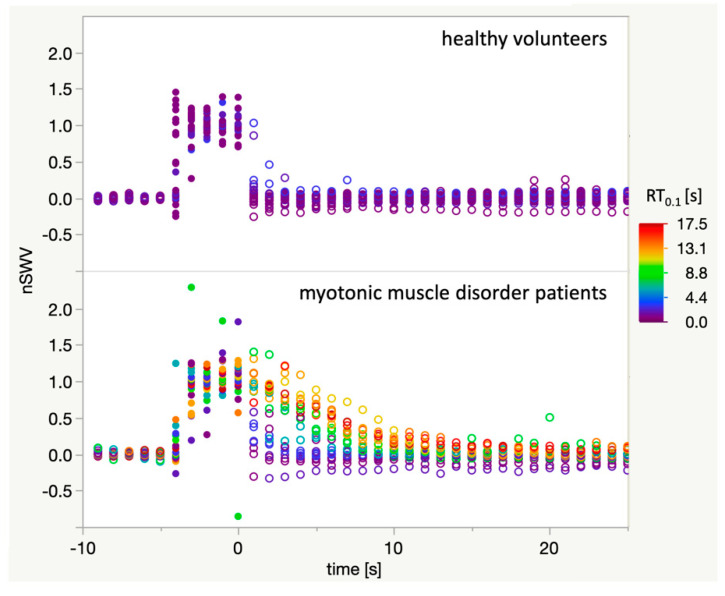
Plot of all 46 acquired imaging sequences of sufficient quality. Open circles: Baseline and relaxation phase, closed circles: fist clenching phase. For each imaging sequence, all data points are coloured according to the respective relaxation time RT_0.1_ with the colour-scale ranging from 0 s (blue) to 18 s (red). It can be appreciated that RT_0.1_ adequately reflects the delayed muscle relaxation after fist clenching as captured with the SWE technique.

The relaxation time RT_0.1_ was determined for each imaging sequence. Figure 4 illustrates that imaging series acquired in myotonic muscle disorder patients where nSWV values only return to baseline after several seconds are indeed assigned a higher RT_0.1_ than others in patients or in healthy volunteers.

**Figure 5:**
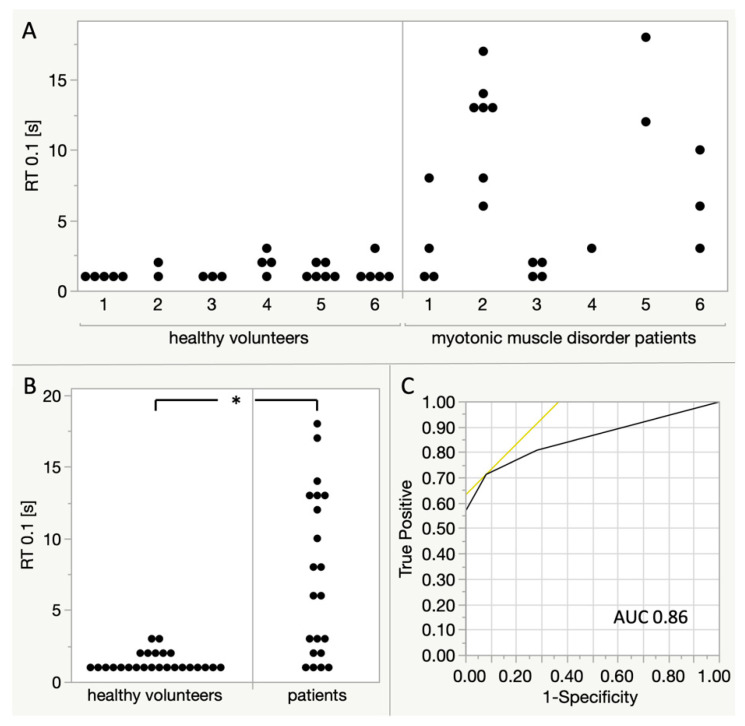
Comparison of RT_0.1_ between myotonic muscle disorder patients and healthy volunteers. Each dot represents one ‘imaging sequence’ and fist clenching manoeuvre, respectively. (**A**) Comparison between individual participants. Patients 1,2: myotonic dystrophy type 1; patients 3–4: myotonic dystrophy type 2, patient 5: paramyotonia congenita, patient 6: genetic testing ongoing. (**B**) Comparison of all measurements between healthy volunteers and patients, Wilcoxon rank sum test shows a statistically significant difference (*p* < 0.0001). (**C**) Receiver operating characteristic (ROC) curve for the distinction of healthy volunteers and patients according to a single RT_0.1_. For a threshold of RT_0.1_ = 3, there is a sensitivity of 0.71 and a specificity of 0.92. The area under the curve (AUC) is 0.86.

RT_0.1_ was highly variable between patients and also between individual imaging sequences in each patient (Figure 5A). With only a low number of measurements, no statistical tests for these comparisons were calculated. Overall, the relaxation time RT_0.1_ was significantly higher (Wilcoxon rank sum test) in myotonic muscle disorder patients (median 6 s, mean 7.38 s, SD 5.72 s) than in healthy volunteers (median 1 s, mean 1.36 s, SD 0.64 s) as shown in Figure 5B. For the differentiation of patients and healthy volunteers according to a single RT_0.1_, the receiver operating characteristic (ROC) area under the curve (AUC) is 0.86, with a sensitivity of 0.71 and a specificity of 0.92 at a threshold of RT_0.1_ = 3 s.

There was no statistically significant difference between patients and healthy volunteers overall in rSWV_mean baseline phase_ and rSWV_mean fist clenching phase_ (Supplemental Figure S3).

## 4. Discussion

Myotonia is the characteristic symptom of hereditary myotonic muscle disorders, causing significant disability [2]. There is a need for quantitative, objective, and non-invasive methods for the evaluation of myotonia severity to aid in diagnosis (for instance, differentiation from muscle cramps), to advance the understanding of natural history and pathophysiology of myotonic muscle disorders and to monitor therapeutic effects in clinical settings or controlled trials.

Here, we present proof of principle that ultrasound shear wave elastography (SWE) can objectively quantify delayed muscle relaxation in myotonia. We determined relaxation time in the flexor digitorum superficialis as measured by SWE to be significantly higher in a small group of patients with different myotonic muscle disorders and clinically evident myotonia (mean 7.38 s in 21 measurements in 6 patients) than in healthy volunteers (mean 1.36 s in 14 measurements in 4 volunteers).

### 4.1. Methodological Considerations of SWE in Muscle

SWE techniques allow the measurement of shear wave velocity parallel to the transducer surface in the imaging plane. With a number of simplifying assumptions (i.e., an isotropic, purely elastic medium without shear wave dispersion induced by viscosity), Young’s modulus can be estimated as a quadratic function of shear wave velocity (*E* = 3 *ρ* *c*^2^ where *E* is Young’s modulus, *ρ* is density and *c* is the shear wave velocity) [14,26,30]. Muscle tissue is highly anisotropic, violating the above-mentioned assumptions [23]. However, it has been shown experimentally ex vivo [25] that Young’s modulus determined by SWE longitudinally to the muscle fibre orientation correlates well with measures obtained with a materials testing technique [25]. Furthermore, in vivo experiments have supported the notion that longitudinal muscle Young’s modulus as assessed by SWE is linearly related to active and passive muscle force [24,31]. SWE has therefore been proposed as a novel method for the quantitative measurement of forces exerted on (or produced by) individual muscles. Our work applies this idea to the time-dependent measurement of finger flexor force in myotonia.

SWE measurements during muscle activation and relaxation were performed before in studies on the force–elasticity relationship in muscle, but during much slower controlled force ramps [31] or without investigating the temporal response [32]. To our knowledge, a quantitative assessment of the speed of muscle relaxation using ultrasound elastography has not been described to date.

In line with other authors’ recommendations [28,29], we did not calculate Young’s moduli based on the measured shear wave velocities due to the possible imprecisions and assumptions implicated. Hence, actual muscle force is likely not linearly, rather quadratically related to SWV values reported by us. Considering other methodical limitations, we expect the impact of using SWV instead of Young’s modulus on the estimation of relaxation times to be negligible.

In our small group of patients with different myotonic muscle disorders, we did not observe a significant difference in baseline rSWV when compared to healthy volunteers, though the statistical power is obviously very low. Indeed, structural changes as expected in myotonic dystrophies might have an impact on muscle elasticity—in Duchenne muscle dystrophy and in inflammatory myopathies, differences have been observed using SWE [19,21,22,28]. Furthermore, although patients and healthy volunteers in our study overall have a comparable mean age, participants were not age-matched individually. Considering a possible age-dependence of absolute muscle elasticity as determined by SWE, there appears to be inconclusive evidence [33,34,35]. In any case, we assume that for the method described in this study using normalization of absolute SWV, changes in baseline elasticity do not substantially impact the assessment of myotonia and relaxation time.

### 4.2. Comparison to Mechanical Dynamometry

In estimating muscle force upon hand-grip in myotonia, our SWE-based approach closely resembles mechanical testing with dynamometers. Isometric hand-grip dynamometers can quantitatively measure the force exerted by finger flexors with high temporal resolution. With this technique, the increased relaxation time after hand-grip in myotonic muscle disorder patients can be determined reliably, which has been used as an endpoint in clinical studies [8,9,10]. Slightly differing definitions are used, e.g., relaxation time from 90% to 5% of maximal force [8] vs. 100% to 10% of a defined target force [10].

In healthy controls, hand-grip relaxation time as determined by dynamometry has been consistently reported to be below one second: Torres et al. [9] examined 18 healthy volunteers (age range 20–59 years) and found a mean RT100-0 of 0.69 s, range 0.59–0.75 s; Moxley et al. [8] described a RT 90-10 mean of 0.33 s in 17 healthy volunteers with mean age 49 years; Horakova et al. [10] examined 35 healthy volunteers (mean age 46.8 years) and found a RT100-10 mean of 0.17 s (range 0.07–0.27 s). The mean RT_0.1_ in healthy volunteers found in this study is higher, most likely due to the lower temporal resolution of SWE and our definition of RT_0.1_ (first nSWV < 0.1, whereby it is always greater than or equal to one second), as well as other technical circumstances, as discussed in Section 4.5.

Mean relaxation times in myotonic muscle disorders were also reported by Torres et al. [9] in 10 myotonic dystrophy patients (4.05 s), Moxley et al. [8] in 29 DM1 patients (1.77 s), Horakova et al. [10] in 20 DM1 patients (2.96 s) and 25 DM2 patients (0.4 s), and Statland et al. [36] in 30 chloride channel mutation (1.74 and 0.74 s; first and sixth sequential handgrip, respectively) and 31 sodium channel mutation non-dystrophic myotonia patients (0.77 s and 1.13 s; first and sixth handgrip, respectively). There is a high variability between individual measurements and between patients.

Similar to the published dynamometry data, we observed a high variability between individual measurements. However, relaxation time as measured by SWE in this study of myotonic muscle disorder patients was higher (mean 7.38 s, SD 5.72 s) than in the studies cited above, which is likely explained by the fact that we explicitly included only patients with clinically apparent myotonia. It would also be conceivable that SWE is more sensitive to increases in muscle force with the result of longer relaxation times.

In comparison between different myotonic muscle disorders, hand-grip myotonia is known to be only mild in myotonic dystrophy type 2 (DM2) [10]. Accordingly, myotonia is thought to have little clinical relevance in this condition [37]. Two of our six patients have genetically confirmed DM2; however, due to the exploratory nature of this study with a low number of patients and measurements, we were not able to perform meaningful comparisons between patients with different diagnoses.

SWE measurements likely reflect individual muscle force, whereas dynamometry can only record the sum of all muscle and joint forces exerted on a limb [24]. In myotonic dystrophy patients, active movements ‘accelerating’ the grip release (i.e., finger extension and thumb abduction) have been observed as well as forearm pronation, ulnar deviation of the wrist, or extension of the wrist [9]. Torres and co-workers remarked that these features persisted even when patients were instructed to merely passively relax after hand-grip, so that they may be hypothesized to constitute a feature of myotonia itself. SWE, in principle, should not be affected by antagonist muscle activity that accelerates agonist muscle stretching and thus would uncover a potential confounder of dynamometry.

### 4.3. Electrodiagnostic Tests in Relaxation Time Measurement

Surface electromyography (EMG) of finger flexors with high-gain amplifiers can also detect the delayed relaxation in myotonic muscle disorders [38]. Leyburn and Walton noted that the relaxation time as recorded by EMG was about 50 percent longer than observed clinically, but that both measures correlated well [39]. Motor nerve stimulation instead of voluntary contraction would also be able to ensure tightly controlled muscle activation, as it is feasible in parallel with EMG and force dynamometry [40]. Hand grip dynamometry without EMG correlation has been considered valid for the assessment of myotonia and used as an endpoint in controlled clinical trials [4,5,11]. Considering the methodological similarity of SWE with dynamometry, we propose that SWE can also provide a valid assessment of myotonia.

### 4.4. Technical Challenges of SWE in Hand-Grip Myotonia

The reliability of ultrasound SWE as applied in this work is limited due to technical issues. As described in the results section, in an examination time of 30–45 min for each participant, the number of acquired imaging sequences of sufficient quality varied considerably, which is attributed to the time necessary for initial transducer positioning and the repetition or later exclusion of imaging sequences when inadvertent transducer movement occurred during a fist-clenching manoeuvre.

The flexor digitorum superficialis muscle forms multiple tendons and is tightly embedded between other flexor muscles of the forearm. Individual constitutional or, in the case of myotonic dystrophy patients, pathological differences (i.e., thickness of subcutaneous fat, relative muscle hypertrophy or atrophy, muscle echogenicity) affect the ease and speed of transducer positioning, considering that a precise longitudinal orientation in relation to the muscle fibres is required [23,29], as well as stability of the imaging plane during and after the fist-clenching manoeuvre.

Due to the low repeatability of a single measurement attempt, we were unable to adequately assess the warm-up phenomenon as in dynamometry studies [41].

It may be argued that the shear wave velocity measurements during muscle activation and early muscle relaxation in this study are confounded by movement of the structures imaged. As we did not perform an isometric hand-grip exercise but simply asked participants to clench their fist, shortening of the flexor digitorum muscles occurs. Passive muscle shortening on its own, contrarily to the effect observed here, has been shown to correspond to decreased longitudinal muscle elasticity and thus decreased shear wave velocity [23,25].

Otherwise, it is conceivable that the ultrasound imaging plane is displaced from the flexor digitorum superficialis into different muscles or tendinous structures with a higher elasticity and thus higher shear wave velocity. We observed such displacement frequently during the fist-clenching phase, and while we did not observe a significant difference in rSWV between groups, these measurements need to be interpreted cautiously. During the relaxation phase, if out-of-plane movement was observed, it generally resulted in a failure of SWV measurements to return to baseline entirely. For this reason, we defined a return to baseline values as a quality indicator and requirement for inclusion for data analysis. An isometric hand-grip task may be able to partly mitigate the former issues, though some relative muscle movement of the flexor muscles is still to be expected due to tendon elasticity and muscle bulging [42,43]. A transverse imaging plane forearm section would be robust against lateral muscle movement, but it appears questionable whether muscle SWE is meaningful in a transverse plane due to the tissue’s mechanical anisotropy [23,25]. Measurements in parallel to the muscle fiber orientation are generally recommended [44].

Technical improvements such as devices for immobilization of the participants’ arm and hand (as employed in some dynamometry studies [9,36]) and stabilization of the ultrasound transducer may be able to improve reliability of ultrasound SWE for the quantification of hand-grip myotonia.

### 4.5. Temporal Resolution of SWE

With one measurement per second, the temporal resolution of SWE as applied in this work limits its ability to differentiate between healthy subjects and less severe cases of myotonia.

This is due to two factors: on the one hand, in protocols relying on voluntary muscle activation as in ours, the accuracy of timing depends on how quickly participants process verbal instructions. In testing with hand-grip dynamometers, the start of the relaxation phase can still be determined precisely; in our approach, we relied only on a fixed sequence of instructions synchronous to ultrasound imaging—which introduces an imprecision of roughly one second (as apparent in Figure 4 upon the start of the fist-clenching phase and the relaxation phase respectively in healthy volunteers). A similar observation made by Sasaki et al. [32]—who performed simultaneous SWE and force measurement during electrical stimulation—highlights that the delay caused by SWE signal processing induces a phase shift. The imperfect synchronization is underlined by that fact that our exploratory ROC curve analysis yielded a threshold of 3 s for RT_0.1_, much longer than expected relaxation times in healthy volunteers. This technical limitation could be partly overcome, for example, with simultaneous dynamometry.

On the other hand, a temporal resolution of one second is fundamentally insufficient to differentiate between RT in healthy volunteers (consistently below 1 s when determined by mechanical dynamometry, as cited above in Section 4.2) and patients with some myotonic muscle disorders with RTs as low as 1–2 s such as DM2 (cited above as well). While we are not aware of clinical ultrasound systems currently capable of SWE with a significantly higher frame rate, this is not a physical limit. Shear wave generation, propagation, and data acquisition last approximately 30–35 milliseconds, resulting in a theoretical boundary of 20–30 frames per seconds. Performance has been limited by acoustic power safety considerations as well as data-processing [26,30]. Other techniques not based on acoustic radiation force based shear wave generation may accomplish even higher frame rates in 2D ultrasound SWE [45].

So far, in many clinical applications of ultrasound SWE, time averaging is performed for noise reduction as temporal resolution is secondary [14]. In contrast, this work highlights the utility of the relatively high frame rate achievable by ultrasound SWE in a neuromuscular setting when compared to magnetic resonance elastography (MRE), for example.

### 4.6. Study Limitations

The main limitations of this work include the low number of participants and measurements. Furthermore, patients and healthy volunteers were not matched for age and gender. Various technical challenges exist in the acquisition of high-quality SWE measurements which are discussed in detail above. Blinding was not feasible because of the obvious clinical characteristics of patients. Intra- and interrater re-test reliability were not assessed; however, this would be complicated by the fact that dynamometry studies also have shown a high variability of relaxation time measurements. Whilst most patients had a genetically or electrophysiologically confirmed diagnosis of a myotonic muscle disorder, we did not perform direct correlation of SWE with other means of myotonia assessment such as dynamometry or electromyography. Still, we interpret the large difference apparent between groups as a sufficient ‘proof-of-principle’ that ultrasound SWE can measure myotonia in a clinical setting and we are planning further measurements to address the issues discussed.

### 4.7. Conclusions and Outlook

In this work, we aim to demonstrate that the relaxation time of finger flexor muscles as a measure of myotonia can be quantitatively determined using ultrasound SWE. Thereby, we propose ultrasound SWE as a widely available, non-invasive technique for assessment of myotonia in general to aid in diagnosis and monitoring of therapeutic effects. In contrast with techniques employed to date, ultrasound SWE provides an insight into individual muscle mechanics. The observation and quantification of myotonia in proximal or truncal regions—where clinical observation and mechanical measurement of myotonia are more difficult—might be rendered possible by this technique [46,47]. In broader terms, this work illustrates the practical utility of the dynamic, time-resolved mode of operation of ultrasound SWE. It highlights its potential as part of neuromuscular ultrasound, a rapidly evolving diagnostic modality in neuromuscular medicine.

## Figures and Tables

**Table 1:** Patient characteristics. Patients with a myotonic syndrome were recruited for the study from our outpatient clinic for muscle disorders.

Patient	Gender	Age [Years]	Diagnosis	Anti-Myotonic Medication
1	female	22	myotonic dystrophy type 1	none
2	female	53	myotonic dystrophy type 1	none
3	male	38	myotonic dystrophy type 2	lamotrigine 250 mg/d
4	male	35	myotonic dystrophy type 2	none
5	female	35	paramyotonia congenita	magnesium as needed
6	male	28	marked myotonic discharges on needle EMG, whole exome sequencing for myotonia congenita and paramyotonia congenita negative, testing for myotonic dystrophy ongoing	lamotrigine 200 mg/d

## Data Availability

The data presented in this study are available on request from the corresponding author.

## Supplementary Materials

The following are available online at https://www.mdpi.com/2075-4418/11/2/163/s1, Figure S1: Example SWE imaging sequences with insufficient measurements quality, Figure S2: Two representative imaging sequences in myotonic muscle disorder patients, Figure S3: Comparison of mean baseline raw shear wave velocities (rSWV) and fist clenching phase rSWV between patients and healthy volunteers, Video S1: Clinical examination of hand-grip myotonia in one patient.
