# Supplementary material for: Muscle Ultrasound Shear Wave Elastography as a Non-Invasive Biomarker in Myotonia
Source: Diagnostics (Basel). 2021 Jan 23;11(2):163. doi: 10.3390/diagnostics11020163 (PMC7911703; doi:10.3390/diagnostics11020163)
Supplement: Supplementary file 1 [file diagnostics-11-00163-s001.zip › supplementary_files/supplementary_figures_kronlage_SWE_myotonia_revision.pdf]

Supplementary Figures

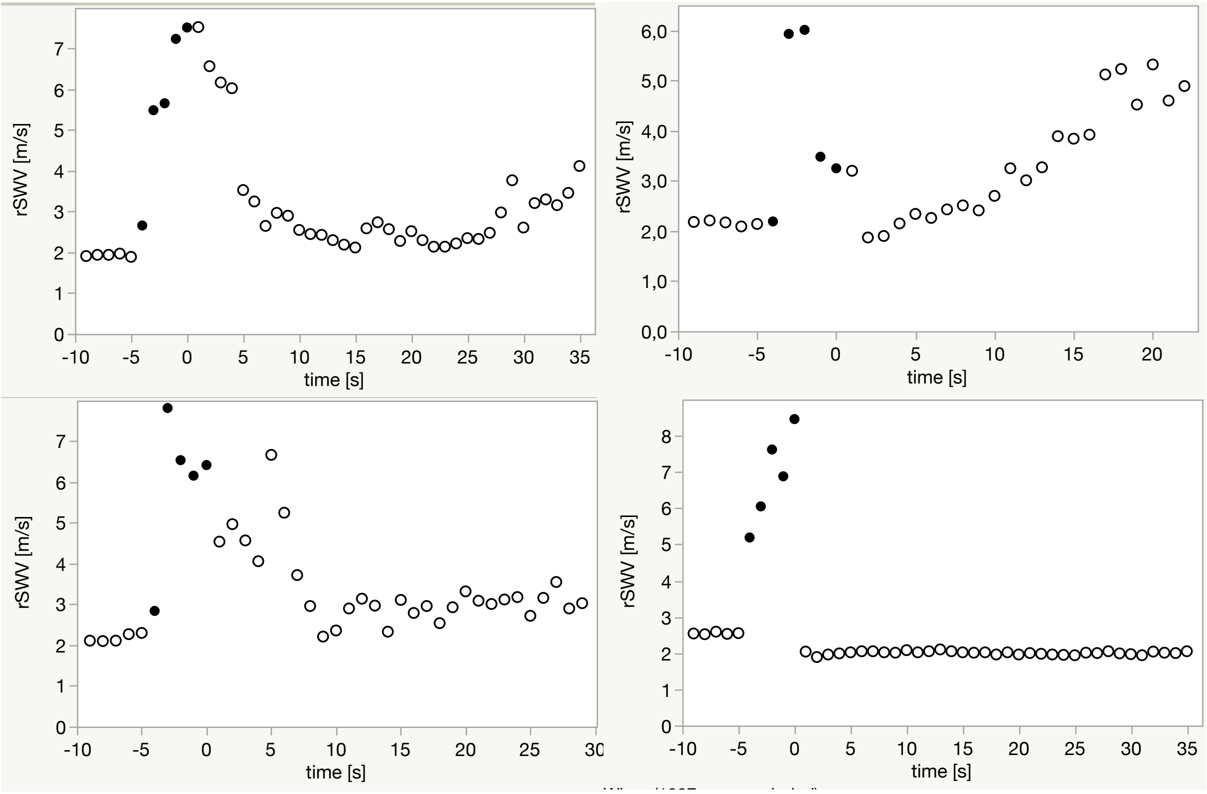

Supplemental Figure S1: Example SWE imaging sequences with insufficient measurement quality. In the relaxation phase, there is no return to baseline shear wave velocity values even after extended observation periods. We attribute this to small unavoidable displacements of the hand-held ultrasound transducer during the fist-clenching manoeuvre.

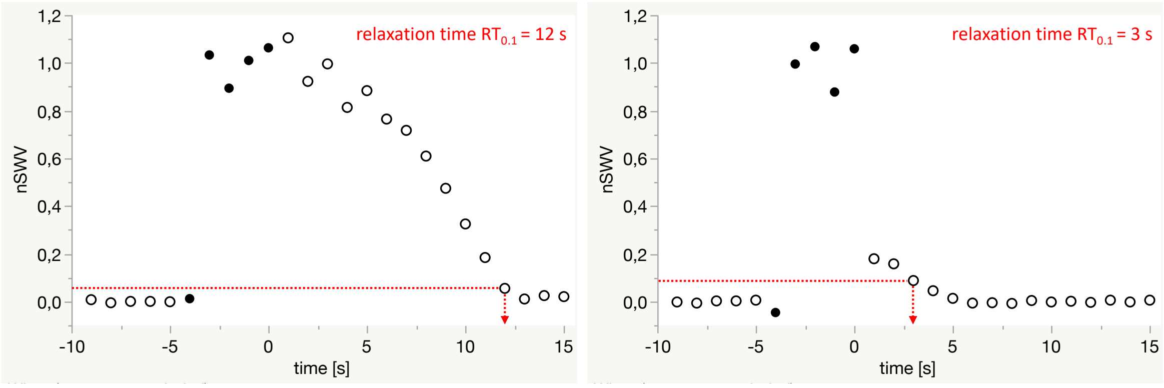

Supplemental Figure S2: Two representative imaging sequences in myotonic muscle disorder patients. Normalized shear-wave velocities (nSWV) on the y-axis plotted against time in seconds. The relaxation time $RT_{0.1}$ is defined as the time of the first measurement in the relaxation phase where nSWV < 0.1. This is illustrated graphically for the two example imaging sequences, yielding a $RT_{0.1}$ of 12s in the first and 3s in the second case.

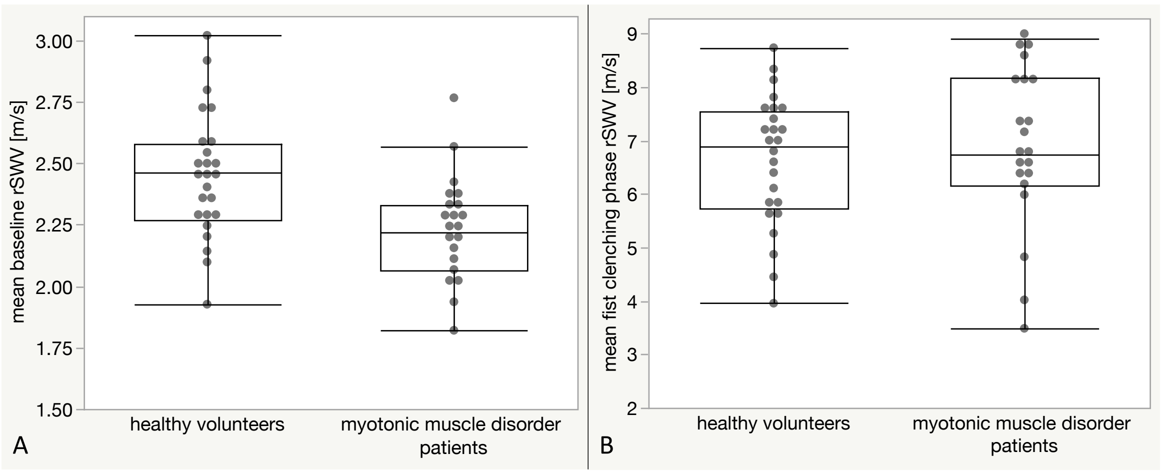

Supplemental Figure S3: Comparison of mean baseline raw shear wave velocities (rSWV) and fist clenching phase rSWV between patients and healthy volunteers. Dot plots and corresponding box plots of each imaging sequence. Wilcoxon rank sum test showed no statistically significant differences between groups (p>0.05).
